# Economic incentives contribute little to reducing agricultural damage from invasive non‐native species: evidence from raccoon management in Hokkaido, Japan

**DOI:** 10.1002/ps.70397

**Published:** 2025-11-23

**Authors:** Kota Mameno, Takaaki Suzuki, Saya Yamaguchi, Mayumi Ueno, Takahiro Kubo

**Affiliations:** ^1^ Research Faculty of Agriculture Hokkaido University Sapporo Japan; ^2^ Research Center for Wildlife Management Gifu University Gifu Japan; ^3^ Research Institute of Energy, Environment and Geology Hokkaido Research Organization Sapporo Japan; ^4^ Research Faculty of Humanities and Human Sciences Hokkaido University Sapporo Japan; ^5^ Biodiversity Division National Institute for Environmental Studies Tsukuba Japan; ^6^ Graduate School of Agriculture Hokkaido University Sapporo Japan; ^7^ Geography and the Environment University of Oxford Oxford UK

**Keywords:** agricultural damage, citizen support, invasive non‐native species, impact evaluation, null result

## Abstract

**BACKGROUND:**

An economic incentive scheme is utilized to enhance citizens' support for managing invasive non‐native species. However, the effectiveness of the incentive scheme in the outcomes of the management remains unclear. This study investigates the effectiveness of economic incentives in managing invasive non‐native species, focusing on their impact on reducing crop damage. Using data from raccoon management in Hokkaido, Japan, and the Japanese agricultural census, our analysis applies an inverse probability‐weighted regression adjustment to evaluate the outcomes.

**RESULTS:**

The findings reveal that economic incentives for capturing raccoons do not significantly reduce crop damage. Additionally, although these incentives increase the number of captured raccoons, the additional captures do not result in measurable reductions in agricultural damage.

**CONCLUSION:**

The absence of positive results underscores the need to develop and evaluate evidence‐based management strategies for invasive non‐native species. This study recommends implementing outcome‐based incentive schemes that align rewards with measurable management goals. Additionally, testing and refining the design of incentive schemes based on their actual effects on management outcomes remain critical. Despite the null results, this study provides valuable insights into designing incentive schemes that garner citizen support while avoiding redundancy. These findings contribute to advancing effective management strategies for invasive species. © 2025 The Author(s). *Pest Management Science* published by John Wiley & Sons Ltd on behalf of Society of Chemical Industry.

## Graphical abstract

This study reveals the nonsignificant effects of economic incentives reducing crop damage by raccoons. Our results also show that while these incentives increase the number of captured raccoons, the additional captures fail to result in measurable reductions in agricultural damage.

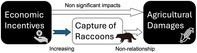

## 1 INTRODUCTION

Invasive non‐native species (INNS) pose a significant threat to ecosystems and human well‐being.^1^, ^2^ They have negative impacts on food security derived from agricultural damages, as well as biodiversity conservation.^3^ For instance, INNS caused an estimated US$278 million in agricultural losses in Japan from 2000 to 2017,^4^ in addition to US$36 billion in the EU^5^ and US$510 billion in the US.^6^ Effective INNS management is essential for the sustainability of agriculture and preserving biodiversity,^7^ protecting human health,^8^ promoting well‐being^9^ and supporting local economies.^10^ Despite its importance, only limited INNS management efforts have been successful, and much of the current management is insufficient to mitigate global impacts.

One major challenge in INNS management is ensuring sustained and widespread public engagement and commitment.^11^, ^12^ Effective INNS management requires sustained resources and long‐term efforts. Citizen involvement is critical to achieving success in these efforts. Indeed, many successful INNS management cases have relied on the support of local stakeholders.^13^ The Intergovernmental Science‐Policy Platform on Biodiversity and Ecosystem Services (IPBES) and the Convention on Biological Diversity (CBD) recommend involving citizens as key players in managing INNS to address resource shortages.^2^, ^14^

Economic incentives, including bounties and subsidies, are widely used as tools to engage citizens in the management of environmental resources, including pest and INNS management.^15^, ^16^ A growing body of literature has examined the causal impact of economic incentives on environmental resource management and conservation. In the agricultural sector, substantial evidence supports the effectiveness of economic incentives such as payment for ecosystem services (PES) and agro‐environmental Schemes.^17^, ^18^, ^19^ For example, economic incentives have been shown to promote environmentally friendly land use, such as reducing grazing pressure from livestock, with continued effects even after the incentives are withdrawn.^17^ However, these incentives often involve trade‐offs, such as enhancing conservation practices, whereas yields decrease.^20^ In the biological conservation sector, increasing awareness of the causal revolution^21^ has led to studies evaluating the impact of PES on biodiversity conservation and wildlife management.^22^, ^23^ One such study found that unequal payment distributions reduce conservation efforts, and that large payment amounts do not necessarily increase those efforts.^24^ These findings underscore the importance of carefully designing environmental policies that use monetary incentives to effectively drive behavioral change.

In the context of INNS management, although the importance of economic incentives is recognized, much of the discussion has focused on valuation studies that assess the costs of INNS to properties or the value of managing INNS.^7^, ^25^ A few theoretical and bio‐economic model studies have also been conducted.^26^ Despite recognizing the importance of evaluating incentive‐based tools,^27^ empirical evidence of the effects of economic incentives on INNS management outcomes remains limited. Consequently, the effectiveness of providing economic incentives as a strategy for managing INNS remains unclear.

In order to address these gaps, this study examines the impact of economic incentives for capturing INNS on reducing agricultural damage, a key outcome of INNS management. The study focuses on raccoon management in the Hokkaido Prefecture, Japan, where raccoons are listed among the 100 Worst Invasive Alien Species in Japan.^28^ Despite being one of the most intensively managed species, raccoon populations continue to expand in the region. Accordingly, our specific objective is to quantify how bounties for raccoon capture affect the reduction of agricultural damage.

This study makes two significant contributions to the literature. First, it quantifies the causal effect of economic incentives on INNS management. Given the importance of efficiently using resources in INNS management, this study provides valuable insights for policymaking aimed at achieving optimal outcomes. Furthermore, impact evaluation methods have gained attention as tools to improve wildlife management and biodiversity conservation.^21^, ^29^ Second, this study shifts the focus of evaluation from the number of captured raccoons to agricultural damage reduction, which is the primary goal of policy. In recent decades, outcome‐based incentive schemes have been discussed primarily in agri‐environmental contexts to improve efficiency (e.g.^30^, ^31^). However, discussions of outcome‐based incentive schemes in invasive species and wildlife management are limited. This study not only evaluates the impact of incentive schemes on agricultural damage outcomes, but also advocates the development of outcome‐focused incentive schemes in the management of INNS.

### 1.1 Background of raccoon management and the policy setting in Japan

Raccoons were introduced to Japan through the abandonment of pets and animals imported for commercial purposes, leading to significant damage to native ecosystems and property. They cause economic losses in agriculture and threaten biodiversity, such as preying on the Japanese crayfish (*Cambaroides japonicus*), an endemic and endangered species in Japan.^32^ Raccoons also pose a public health risk by spreading zoonotic diseases such as the raccoon roundworm (*Baylisascaris procyonis*).^33^, ^34^ In 2022, agricultural losses in Japan due to raccoons exceeded 450 million JPY (≈US$3 million).^35^ To address these impacts, raccoons were designated as invasive alien species under the Japanese Invasive Alien Species Act in 2005, leading to the development of nationwide management programs. Moreover, given the huge agricultural damages, trapping of raccoons has been undertaken as pest control with support from various government agencies. The government also has introduced support measures including financial incentives, under the Act on Special Measures for Prevention of Damage Related to Agriculture, Forestry, and Fisheries Caused by Wildlife.^36^ Local governments have implemented additional support strategies. Although raccoon management has provided multiple public and private benefits, this study focuses on the role of economic incentives in reducing agricultural damage, which is the main policy objective related to financial support.

**Figure 1:**
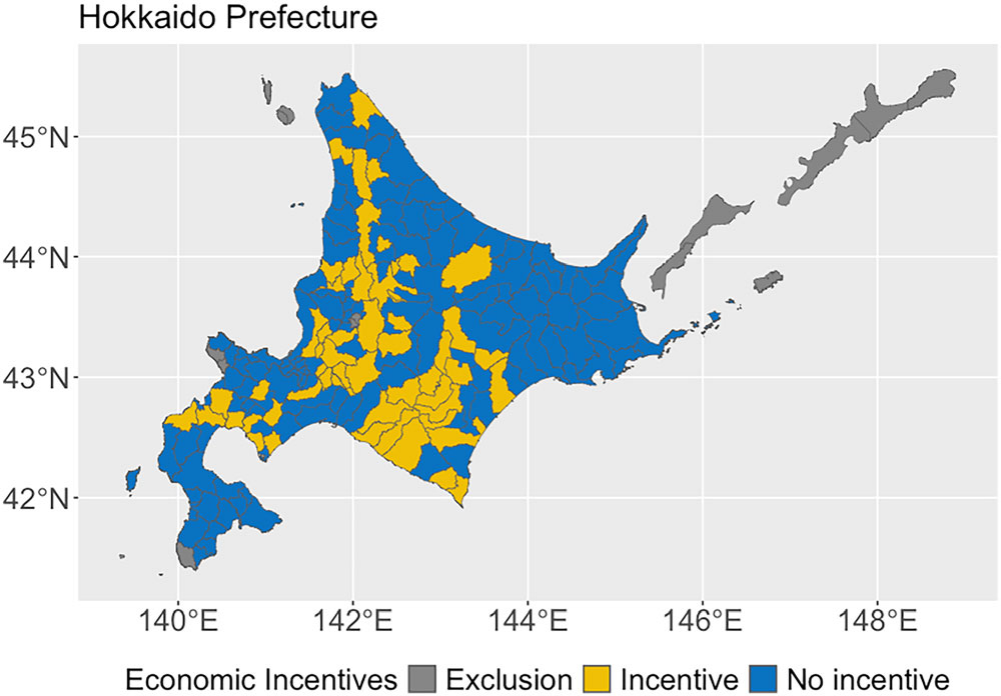
Study site in Hokkaido prefecture and utilization of economic incentives in 2017. Excluded cities have no farmland data in the census.

Hokkaido Prefecture, where our study is based (Fig. 1), has experienced notable raccoon colonization and population growth owing to omnivorous feeding habits, high reproductive rates and the absence of natural predators.^37^ Since 1999, the Hokkaido government has been managing raccoon populations through eradication programs. Despite these efforts, raccoon distribution and agricultural damage continue to rise.^38^ In 2022, agricultural damage in Hokkaido reached 144 million JPY (≈US$1 million). Local municipalities have mainly addressed raccoon management through the law designed to measure wildlife damage to agriculture and property. Although the Ministry of the Environment has issued management guidelines and encouraged cooperation with local municipalities and the Ministry of Agriculture, Forestry and Fisheries (MAFF),^39^ the raccoon management strategy remains fragmented. Some municipalities offer economic incentives to citizens to capture raccoons, whereas others do not. A key challenge is the absence of a formal evaluation process for local efforts.^40^ Economic incentives are widely used without evaluation of effectiveness in raccoon management, which can lead to inefficient resource allocation. Our study on the impact of economic incentives provides valuable insights into optimizing raccoon management, particularly with limited resources.

## 2 MATERIALS AND METHODS

### 2.1 Identification strategy

Our primary objective was to estimate the impact of economic incentives on reducing raccoon‐related crop damage as part of INNS management. Specifically, we calculated the average treatment effect on the treated (ATET), which measured the average effects of economic incentives on economic losses from raccoon crop damage in the municipalities that provide economic incentives. Following previous studies,^41^, ^42^ ATET is defined using potential outcomes (Y) and the economic incentive treatment, $T\in \left\{0,1\right\}$, which is 1 if municipalities i implements economic incentive schemes, and 0 otherwise, as follows:

$$\text{ATET}=E\left\{Y_{i1}-Y_{i0}|T_{i}=1\right\}=E\left(Y_{i1}|T_{i}=1\right)-\left(Y_{i0}|T_{i}=1\right)$$

As counterfactual effects (i.e. outcomes under different treatment statuses; in this case, outcomes in the treatment area had no incentives been provided) cannot be directly observed, this study uses inverse probability weighting with a regression adjustment (IPWRA) estimator to estimate causal effects by addressing missing data.^43^, ^44^ One key advantage of IPWRA is its double robustness. The use of economic incentives may be influenced by factors such as landowners' characteristics, conservation intentions and the socio‐economic conditions of agricultural communities, including budgets for INNS management.^39^, ^45^ Therefore, selection bias associated with the treatment variable—economic incentive use—must be addressed to assess its effects. Propensity score matching methods are commonly used to control for selection bias, but these rely on the strong assumption that the treatment model is correctly specified. By relaxing this assumption, IPWRA provides consistent and unbiased treatment effect estimates, as long as the treatment or outcome model is correctly specified.^43^, ^44^ The IPWRA method involves two stages: in the first stage, the propensity score is estimated, and in the second stage, inverse probability‐weighted linear least squares estimates are produced. Combining the conditional mean model with the propensity score model yields a doubly robust estimator for the ATET. The treatment model is derived from the inverse probability‐weighted propensity scores for treatment and is expressed as follows^46^:

$$\hat{p}\left(X\right)=\mathrm{Pr}\left(T_{i}=1|X\right)=E\left(T_{i}|X\right)$$

where X is a vector of observed municipality‐level variables that influence treatment. In this study, X includes agricultural characteristics, conservation status, and raccoon management status including catch per unit effort (CPUE; Table 1). A pseudo‐sample, which mimics a random treatment assignment, is estimated based on the propensity scores. The treatment model is then described by combining the regression adjustment model with inverse probability weighting as follows:

$$\text{ATET}=N_{1}^{-1}\sum_{i=1}^{N}\left[\left(\alpha_{1}+X\beta_{1}\right)-\left(\alpha_{0}+X\beta_{0}\right)\right]=\left(\hat{\alpha_{1}}-\hat{\alpha_{0}}\right)+\bar{X_{1}}\left(\hat{\beta_{1}}-\hat{\beta_{0}}\right)$$

where α and β are parameters, and ${\bar{X_{1}}=N}_{1}^{-1}\sum_{i=1}^{N}T_{i}X_{i}$ is the average over the subsample that introduces economic incentives. Our analysis was conducted using the ‘teffects’ command in Stata 19.

**Table 1:** Key background characteristics of the cities

	Does the municipality utilize economic incentives?		
Variables	Yes (Y)	No (N)	Diff.
	Mean (SD)	Mean (SD)	(Y—N)
Cost of crop damage per farmland area (JPY)	0.241 (0.422)	0.372 (1.471)	−0.132
Number of captured raccoons	147.792 (168.923)	58.407 (97.059)	89.386***
Have raccoons been sighted? (1 = Yes, 0 = No)	0.981 (0.137)	0.585 (0.495)	0.396***
Number of farmers	256.774 (221.674)	175.432 (150.763)	81.341**
Number of full‐time cultivators	189.415 (168.012)	129.297 (122.736)	60.118**
Number of certified superior farmers; they can obtain focused support measures	200.283 (182.344)	132.449 (128.636)	67.834**
Number of farmers with the highest proportion of agricultural sales from fruits	1.604 (3.885)	4.364 (23.166)	−2.761
Number of farmers with the highest proportion of agricultural sales from vegetables	21.849 (36.173)	12.932 (25.263)	8.917
Number of agri‐communities that have meetings about nature resource conservation	27.717 (26.918)	17.847 (15.921)	9.870**
Number of agri‐communities that have meetings about agricultural production	26.547 (28.020)	16.093 (15.830)	10.454**
Number of agri‐communities that have meetings about irrigation canal and reservoir management	25.434 (27.249)	14.136 (13.819)	11.298*
Number of agri‐communities that conserve farmland	18.189 (20.459)	12.076 (15.109)	6.112***
Number of agri‐communities that conserve forest areas	3.019 (5.706)	3.008 (5.349)	0.010***
Number of agri‐communities that conserve rivers and channels	13.736 (17.665)	6.729 (10.583)	7.007***
Number of agri‐communities that conserve agricultural canals	20.849 (23.922)	11.398 (13.138)	9.451***
Is there any support for the disposal of dead raccoons? (1 = Yes, 0 = No)	0.585 (0.497)	0.390 (0.490)	0.195**
Is there any support to buy traps? (1 = Yes, 0 = No)	0.038 (0.192)	0.042 (0.202)	−0.005***
Number of traps that the municipality has	26.660 (36.078)	16.466 (32.772)	10.194***
Catch per unit effort (CPUE) of raccoons in 2016	0.083 (0.123)	0.036 (0.080)	0.048**
Did the municipality conduct raccoon capture? (1 = Yes, 0 = No)	0.925 (0.267)	0.568 (0.497)	0.357***

*Note*: The last column shows the results of the two‐tailed Student's *t*‐test for differences in means. ***, ** and * indicate the 1%, 5% and 10% levels of significance. SD, standard deviation.

### 2.2 Mechanism analysis

**Figure 2:**
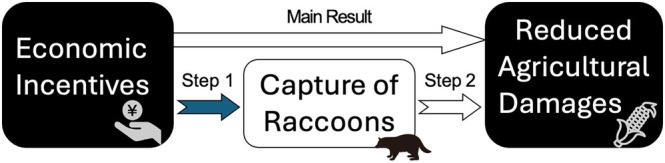
Model of economic incentives reducing agricultural damages. The blue arrow indicates statistical significance.

In the above analysis, null result was obtained; therefore, to identify a bottleneck in the effect of economic incentives on crop damage, we performed an additional analysis. We divided the effects of economic incentives on crop damage into two steps, as shown in Fig. 2: (1) economic incentives might influence the number of raccoons captured and (2) the number of captured raccoons might subsequently influence crop damage. To examine the causal effect of economic incentives on the number of raccoons captured, the same datasets were used, but with the number of captured raccoons as the outcome variable instead of crop damage. Next, a regression analysis was conducted to examine the relationship between the number of captured raccoons and crop damage caused by raccoons. The relationship between these two variables is represented by:

$${\text{CropDamage}}_{i}=\gamma {\text{Capture}}_{i}+\delta X_{i}+\epsilon_{i},$$

where ${\text{CropDamage}}_{i}$ represents crop damage caused by raccoons per unit of farmland area in municipality i, ${\text{Capture}}_{i}$ is the number of captured raccoons in municipality i, $X_{i}$ is a vector of observed municipality i characteristics, including agricultural conditions and raccoon management factors, and $\epsilon_{i}$ is the error term with heteroskedasticity‐robust standard errors.

### 2.3 Data

In order to examine the impacts, multiple data sources were used to create a comprehensive dataset by combining raccoon management records from Hokkaido with agricultural community census data. The raccoon management records, summarized at the municipality level for 2018, were provided by the Hokkaido Prefecture (unpublished data). These records included data on the budget for raccoon management, the number of traps set and raccoons captured, the availability of incentives and other support for capturing raccoons, and economic losses resulting from crop damage by raccoons in 2017, which was the main outcome variable. As control units, data were obtained from the Rural Community Card and the 2015 Japanese Census of Agriculture and Forestry, conducted by MAFF every 5 years since 1950. This census included information on farming activities (e.g. cultivated farmland area and the number of farmers for whom farming is the primary occupation) and environmental and agricultural resource conservation activities at the agricultural community level. In addition to agricultural data, to control for the population of raccoons in each municipality, the dataset also included data on the CPUE of raccoons and economic losses due to crop damage by raccoons in the previous year (2016). These data, derived from the Hokkaido District Agriculture Office's annual pest management survey, slightly differed from the 2017 crop damage data mentioned earlier. All data were aggregated at the municipality level to align with the raccoon management records, with the municipality as the unit of observation. Eight municipalities were excluded from the analysis due to the absence of farmland, leaving 171 observations. As the dataset was provided by the Hokkaido Prefecture and based on agricultural census data, no ethics approval was required for our study. Table 1 shows the differences in background characteristics based on the use of economic incentives for raccoon management. These differences indicate a selection bias in management policy.

## 3 RESULTS

### 3.1 Main results

Table 2 presents the IPWRA estimates of the ATET of economic incentives on agricultural damage caused by raccoons per unit of farmland area. The analysis revealed that economic incentives did not significantly impact agricultural damage. Specifically, the left side of Table 2 shows that, although not statistically significant, crop damage per unit of farmland decreased by 0.194 points in municipalities that provided economic incentives for raccoon management aimed at crop damage prevention. The right side of Table 2, which focuses on municipalities where raccoons have been sighted, confirms these findings. We also verified that the two assumptions necessary for robust results in IPWRA were met, as detailed in the robustness check (Supporting information Appendix S1). As an additional robustness check, we analyzed the total economic support related to raccoon capture, rather than focusing on a single economic incentive for capture. This support included funding for traps and the disposal of dead or captured raccoons. This analysis reaffirmed the null results regarding the impact of economic incentives on reducing crop damage by raccoons (Table S1 in Appendix S2). Furthermore, additional analyses using crop‐specific damage outcomes also show consistently null results across all agro‐products (Table S2 in Appendix S3). Therefore, our results suggested that economic incentives had a minimal effect on preventing agricultural damage caused by raccoons.

**Table 2:** Estimated results in inverse probability weighting with a regression adjustment (IPWRA) estimator: treatment effect on treated (ATET) of economic incentives in reducing agricultural damage caused by raccoons

Variables	Full sample			Subsample (sighted raccoons)		
	ATET	Robust SE	*P*‐value	ATET	Robust SE	*P*‐value
Incentive	−0.194	0.133	0.145	−0.184	0.133	0.166
Number of observations	171			121		

### 3.2 Mechanism

The results in the additional analysis showed that economic incentives positively affected the number of raccoons captured, increasing the capture count by ≈61.3 raccoons (Table 3). The regression analysis revealed that the number of captured raccoons does not significantly affect crop damage (Table 4). These additional results in the mechanism suggested that, whereas economic incentives increased the number of raccoons captured, this did not reduce agricultural damage (Fig. 2).

**Table 3:** Estimated results in inverse probability weighting with a regression adjustment (IPWRA) estimator: treatment effect on treated (ATET) of economic incentives on the number of captured raccoons

Variables	Full sample			Subsample (sighted raccoons)		
	ATET	Robust SE	*P*‐value	ATET	Robust SE	*P*‐value
Incentive	61.339	27.597	0.026	63.035	27.803	0.023
Number of observations	171			121		

**Table 4:** Regression analysis results: the relationship between the number of captured raccoons and agricultural damage by raccoons

	Full sample			Subsample (sighted raccoons)		
Variables	Coeff.	Robust SE	*P*‐value	Coeff.	Robust SE	*P*‐value
Capture	0.0002	0.001	0.719	0.0002	0.001	0.756
Controls	Yes			Yes		
Region dummies	Yes			Yes		
*F*‐statistic	2.260		0.001	2.710		0.000
Number of observations	171			121		

## 4 DISCUSSION

Despite the null results, our findings offer valuable insights into managing invasive species with limited resources. Our consistent results show that economic incentives based on the number of captured raccoons do not reduce agricultural damage caused by raccoons. This suggests a need for alternative measures to address such damage. Further analysis revealed that the number of raccoons captured increased when municipalities used economic incentives; however, the number of captured raccoons did not lead to the reduction of agricultural damage. This result suggests that economic incentives have limited agricultural impact and cannot be considered an effective scheme for reducing agricultural damage.

One possible explanation is that the number of raccoons captured was insufficient to reduce damage, indicating that even with economic incentives, management efforts have not reached a low‐density level. Public tolerance of damage also may influence the outcomes, as previous studies have shown that raccoon management typically occurs only when agricultural damage becomes unacceptable (e.g.^37^, ^40^). Another potential explanation is that the incentives primarily encourage capturing raccoons in areas where and at times when they are easy to catch, regardless of the agricultural damage. These efforts do not effectively contribute to reducing agricultural damage or to overall raccoon population management. Indeed, IPBES^2^ reported that hunting programs often focus on areas and seasons when the target species is most abundant, which may limit the effectiveness of such efforts in reducing environmental impacts. Thus, the economic incentives could promote raccoon captures only in locations and seasons with high capture rates. An alternative explanation for this result is compensatory mortality, a hypothesis suggesting that harvest mortality triggers density‐dependent responses in reproduction, offspring survival and female population growth by reducing competition for resources.^47^, ^48^ Under compensatory mortality, raccoon removal does not lead to a substantial reduction in the overall population within the region. Consequently, bounty programs may fail to incentivize additive mortality, limiting their effectiveness in reducing overall raccoon abundance and associated agricultural damage. Related to this, a substantial reduction in agricultural damage may require the removal of a large number of raccoons. Thus, despite an increase in the number of captured raccoons, this might not be enough to reduce agricultural damage.

Our findings align with previous studies indicating that economic incentives are insufficient for wildlife management^49^ and that recreational and commercial hunting, including bounty‐based hunting programs, does not effectively reduce wildlife populations.^50^, ^51^ Additionally, our findings support the idea that outcome‐based incentive schemes, such as those used in agricultural policies, are more effective.^30^ Therefore, we suggest that economic incentives should focus on reducing agricultural damage, rather than simply the number of raccoons captured.

Our results highlight the need for further research to gain valuable insights for policy development. This study focused on financial incentives for capturing raccoons to reduce agricultural damage; however, it did not consider other forms of financial support, such as subsidies for preventive measures like fencing, owing to data limitations. Given the effectiveness of fences in reducing agricultural damage,^52^, ^53^ future research should examine the causal impact of such preventive support. Another limitation of this study is the evaluation of outcomes. We assessed the impact of economic incentives based on agricultural damage, aligning with the policy objectives. However, because INNS provide various benefits, future research should consider multiple outcomes to evaluate the validity of economic incentives. Moreover, understanding whether mortality is compensatory or additive is critical for wildlife management decisions, such as setting hunting quotas or evaluating pest control measures. Although our analysis revealed noncorrelation between the number of raccoons captured and the extent of agricultural damage, the ecological mechanisms underlying this relationship remain unclear.

Furthermore, our limitations highlight the need for spatiotemporal and long‐term panel data to uncover more detailed causal impacts and heterogeneity, which are essential for effective policy‐making. We were unable to assess the long‐term lag between interventions and outcomes. Although the increased number of captured raccoons has a limited immediate impact on reducing agricultural damage, this increased capture could eventually decrease the population and significantly reduce agricultural damage over time. Previous studies on invasive species management show a time lag before the full effects are realized, often accompanied by unintended consequences owing to complex ecosystems (e.g. ^54^). Therefore, the long‐term effects of economic incentives on agricultural damage should be evaluated using a long difference‐in‐differences method. Long‐term monitoring could not only improve the accuracy of evaluations by enabling control for unobserved heterogeneity through panel data analysis, but also enhance our understanding of how economic incentives function over time. Likewise, detailed spatial data could help reveal regional heterogeneity arising from factors such as crop diversity and variation in the intensity of raccoon impacts. Beyond academic research, detailed spatiotemporal data also play a crucial role in informing management practices. This underscores the importance of collaborative research involving governments and citizens, alongside academic researchers.

## 5 CONCLUSION

The management of INNS is a critical issue, yet resources are quite limited. Evidence‐based policy evaluations are essential for implementing effective measures. This study provides evidence of the effects of economic incentives on INNS management outcomes, focusing on raccoons in Hokkaido, Japan. The IPWRA estimates revealed that economic incentives do not significantly reduce agricultural damage in the short term. However, they do lead to an increase in the number of raccoons captured. These findings highlight the importance of evidence‐based development in INNS management. They also support outcome‐based incentive schemes, which are attracted in agri‐environmental programs. Despite the null results, this study can contribute to more effective INNS management in the agricultural sector by preventing redundancy in both research progress and policy development.

## AUTHOR CONTRIBUTIONS

Kota Mameno: Conceptualization (lead); Data curation (lead); Formal analysis (lead); Methodology (equal); Visualization (equal); Resources (equal); Project administration (lead); Funding acquisition (equal); Writing–original draft (lead). Takaaki Suzuki: Conceptualization (support); Resources (equal); Visualization (equal); Funding acquisition (equal); Writing–original draft (support); Writing‐review & editing (equal). Saya Yamaguchi: Data curation (support); Resources (equal); Writing–review & editing (equal). Mayumi Ueno: Data curation (support); Resources (equal); Writing‐review & editing (equal). Takahiro Kubo: Conceptualization (support); Formal analysis (supporting); Methodology (equal); Visualization (equal); Resources (equal); Writing–review & editing (equal).

## CONFLICT OF INTEREST

The authors declare that they have no known competing financial interests or personal relationships that could influence the work reported in this paper.

## ETHICS STATEMENT

None.

## Supporting information


**Data S1.** Supporting Information.

## Data Availability

The data that support the findings of this study are available on request from the corresponding author. The data are not publicly available due to privacy or ethical restrictions.
