# Supplementary material for: Economic incentives contribute little to reducing agricultural damage from invasive non‐native species: evidence from raccoon management in Hokkaido, Japan
Source: Pest Manag Sci. 2025 Nov 23;82(3):2588–94. doi: 10.1002/ps.70397 (PMC12886171; doi:10.1002/ps.70397)
Supplement: Supplementary file 1 — Data S1. Supporting Information. [file PS-82-2588-s001.docx]

**Appendixes**

**Appendix 1**

A conditional independence assumption is necessary for robust results in IPWRA. This assumption posits that treatment assignment is independent of potential outcomes when conditioned on a set of observed covariates. Based on the over-identification test, we fail to reject the null hypothesis that the covariates are balanced (chi-square (14) = 2.893, Prob > chi-square = 0.999). Additionally, the IPWRA estimator relies on the overlapping assumption that each municipality in the control group must have a positive probability of receiving treatment, once controlled for observed confounders (Crump et al., 2009). Figure S1 presents the estimated density of the predicted probabilities for municipalities managing raccoons, with and without economic incentives. We conclude that the overlap assumption is satisfied, although some municipalities without economic incentives are near the propensity scores of treated municipalities and do not fully overlap with those receiving incentives.

Crump, RK, Hotz, VJ, Imbens, GW and Mitnik, OA, Dealing with limited overlap in estimation of average treatment effects. *Biometrika* **96**(1):187-199 (2009). <https://doi.org/10.1093/biomet/asn055>


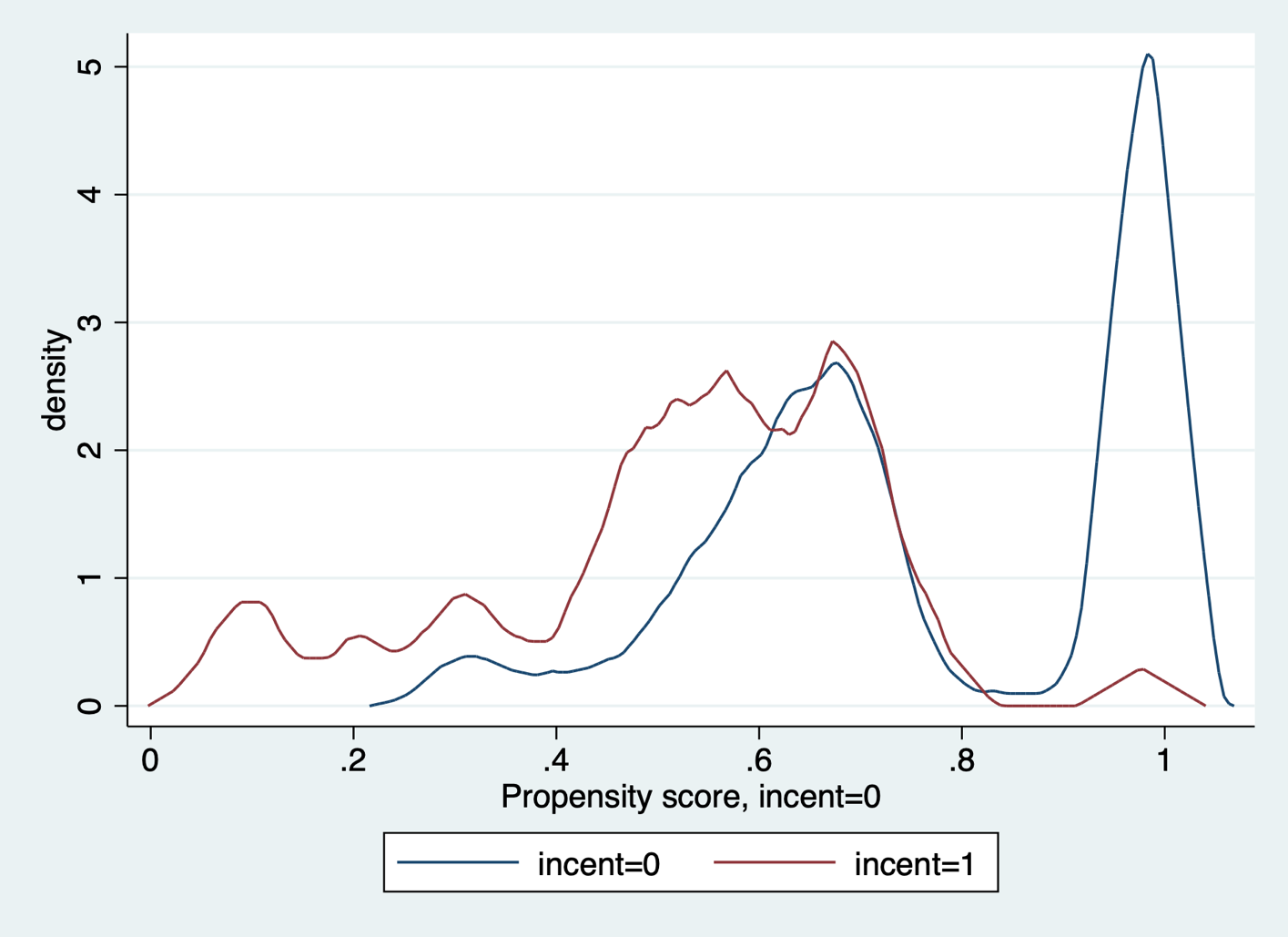


**Figure S1. Overlap assumption test**

**Appendix 2**

**Table S1. Estimated results in inverse probability weighting with a regression adjustment (IPWRA) estimator: the treatment effect on treated (ATET) of total economic support in reducing agricultural damage caused by raccoons.**

|  | Agricultural damages | | |  | Number of captured raccoons | | |
| --- | --- | --- | --- | --- | --- | --- | --- |
| Variables | ATET | Robust  Std. Err. | p-value |  | ATET | Robust  Std. Err. | p-value |
| Incentive | 0.575 | 0.751 | 0.444 |  | 96.509 | 14.509 | 0.000 |
| Number of observations | 171 |  |  |  | 171 |  |  |

**Appendix 3**

**Table S2. Estimated results in inverse probability weighting with a regression adjustment (IPWRA) estimator: treatment effect on treated (ATET) of economic incentives in reducing damage caused by raccoons, focusing on vegetables, grain, fruits, and dairy farming.**

|  | Agricultural damages focusing on vegetables | | | Agricultural damages focusing on grain | | | | Agricultural damages focusing on fruits | | | | Agricultural damages focusing on dairy farming | | | |
| --- | --- | --- | --- | --- | --- | --- | --- | --- | --- | --- | --- | --- | --- | --- | --- |
| Variables | ATET | Robust  Std. Err. | p-value | ATET | Robust  Std. Err. | | p-value | ATET | Robust  Std. Err. | | p-value | ATET | Robust  Std. Err. | | p-value |
| Incentive | 0.0257 | 0.0240 | 0.283 | -0.0701 | | 0.065 | 0.284 | -0.0717 | | 0.0790 | 0.364 | -0.0168 | | 0.0169 | 0.319 |
| Number of observations | 171 |  |  | 171 | |  |  | 171 | |  |  | 171 | |  |  |
